# Association of SIGLEC9 Expression with Cytokine Expression, Tumor Grading, KRAS, NRAS, BRAF, PIK3CA, AKT Gene Mutations, and MSI Status in Colorectal Cancer

**DOI:** 10.3390/cimb46120814

**Published:** 2024-11-29

**Authors:** Błażej Ochman, Anna Kot, Sylwia Mielcarska, Agnieszka Kula, Miriam Dawidowicz, Dominika Koszewska, Dorota Hudy, Monika Szrot, Jerzy Piecuch, Dariusz Waniczek, Zenon Czuba, Elżbieta Świętochowska

**Affiliations:** 1 Department of Medical and Molecular Biology, Faculty of Medical Sciences in Zabrze, Medical University of Silesia, 19 Jordana, 41-808 Zabrze, Poland; d201228@365.sum.edu.pl (B.O.); s85876@365.sum.edu.pl (A.K.); d201109@365.sum.edu.pl (S.M.); s85874@365.sum.edu.pl (D.K.); dorota.hudy@sum.edu.pl (D.H.); 2 Department of Oncological Surgery, Faculty of Medical Sciences in Zabrze, Medical University of Silesia, 41-808 Katowice, Poland; d201070@365.sum.edu.pl (A.K.); d201069@365.sum.edu.pl (M.D.); dwaniczek@sum.edu.pl (D.W.); 3 Department of General and Bariatric Surgery and Emergency Medicine in Zabrze, Faculty of Medical Sciences in Zabrze, Medical University of Silesia, 10 Marii Curie-Skłodowskiej, 41-800 Zabrze, Poland; mszrot@sum.edu.pl (M.S.); jpiecuch@sum.edu.pl (J.P.); 4 Department of Microbiology and Immunology, Faculty of Medical Sciences in Zabrze, Medical University of Silesia, 19 Jordana, 41-808 Zabrze, Poland; zczuba@sum.edu.pl

**Keywords:** colorectal cancer (CRC), human sialic-acid-binding immunoglobulin-like lectin-9 (SIGLEC9), microenvironment, tumor, K-ras oncogene, proto-oncogene proteins B-raf (BRAF), instability, microsatellite

## Abstract

SIGLEC9 (sialic acid-binding Ig-like lectin 9) is a molecule thought to have a significant influence on the immune properties of the colorectal cancer (CRC) tumor microenvironment (TME). In our study, we assessed the expression of the SIGLEC9 protein in CRC tissue and the surgical margin tissue. Using RT-PCR, we analyzed mutations in the KRAS, NRAS, BRAF, PIK3CA, and AKT genes. We observed a significantly elevated expression of the SIGLEC9 protein in CRC tissue compared to the control group. No significant differences were observed in SIGLEC9 protein expression depending on mutations in the KRAS, NRAS, BRAF, PIK3CA, and AKT genes or microsatellite instability (MSI) status. However, we found a significantly higher expression of the SIGLEC9 protein in high-grade tumors compared to the low-grade tumors group. SIGLEC9 expression was significantly associated with the expression of multiple cytokines, chemokines, and growth factors in the CRC TME. These associations suggest the significant potential of SIGLEC9 as a molecule that plays a crucial role in shaping the immune properties of the CRC TME, as well as its potential therapeutic relevance, particularly in the group of high-grade CRC tumors.

## 1. Introduction

SIGLEC9—a member of the sialic acid-binding Ig-like lectin (SIGLEC) family—plays an important homeostatic role in physiological conditions. Normal glycans on epithelial cells in the colon exert immunosuppressive activity, inhibiting the cyclooxygenase-2 expression and PGE2 synthesis in macrophages, acting as immunomodulators in colonic mucosa [1]. SIGLEC9 could also influence VEGF signaling, thus taking part in angiogenic processes [2]. Moreover, gene expression regulation including DNA methylation may also depend on SIGLEC9-related signaling pathways. SIGLEC9 could influence the expression of various genes, regulating immune responses and cell adhesion [3,4]. Eisenberg et al. suggested that SIGLEC9 can act as an immune checkpoint in cancerogenesis because of its immunomodulatory function [5]. Thus, research on the topic could be valuable in the context of developing novel therapeutic strategies in cancer and should be encouraged.

SIGLEC9 belongs to a subfamily of I-type lectins responsible for sialic acid recognition [6]. The protein consists of 463 amino acids and shows homology to other members of the protein family: SIGLEC7 (80%), SIGLEC8 (72%), SIGLEC5 (65%), and CD33 (64%) [3]. Together with SIGLEC5, -6, -7, and -8, it forms a part of a SIGLEC3/CD33-related subset. The V-set N-terminal domain of SIGLEC9 shares the highest number of amino acid residues with other SIGLECs [6]. The protein consists of two C2-set domains, a cytoplasmatic tail with two putative signaling motifs based on tyrosine, a transmembrane domain, and three extracellular, immunoglobulin-like domains. Its extracellular domain was shown to bind α2–3- and α2–6-linked sialic acids [6,7].

Angata et al. found SIGLEC9 expression in granulocytes and monocytes. According to other reports, it is most abundantly present on neutrophils and monocytes, as well as on a smaller population of CD16+CD56− cells. At the same time, weak SIGLEC9 expression was detected on approximately 50% of B cells and NK cells, and small subsets of CD8+ and CD4+ T cells [6,7]. In healthy tissues, strong SIGLEC9 expression was also found in the placenta, spleen, lung, and fetal liver, while the results regarding bone marrow are contradictory [8]. The expression levels of SIGLEC9 have been studied in numerous malignancies, while SIGLEC9 ligand overexpression was detected in various neoplastic cell lines [9]. The molecule seems to perform immunosuppressive functions in the tumor microenvironment (TME), reducing the activity of T cells and decreasing pro-inflammatory cytokine secretion [5]. SIGLEC9 upregulation was observed in tumor-infiltrating lymphocytes (TILs), including CD4+ and CD8+ T cells in colorectal cancer (CRC) [10]. Additionally, sialic acids in stromal cells exerted immunomodulating effects in the TME in the disease [11]. Still, little is known regarding SIGLEC9 expression and function in CRC, or its connections with signaling pathways related to immune responses. Research on the topic is of high importance, as the molecule is a promising target for therapeutic strategies. For example, targeting Sia-SAMPs/Siglec-9 interactions resulted in delayed tumor growth and enrichment in immune infiltrations in CRC [10]. In other malignancies, Siglec-7/9-based CAR T cell therapy [9] or treatment using Siglec-9-based chimeric switch receptors (CSRs) presented prominent results [5].

Other crucial factors shaping the characteristics of the TME in CRC include mutations in the KRAS, NRAS, BRAF, PIK3CA, and AKT genes. The frequency of these mutations varies across studied populations and carries significant predictive and prognostic implications for CRC [12,13]. Research over the past decades on the role of immune checkpoint pathways in tumor progression has led to the development of immunotherapy methods that, when applied to well-defined patient groups, prolong survival and inhibit further disease progression. Current immunotherapy approaches in CRC have been shown to be effective in a small percentage of patients, particularly those with microsatellite instability (MSI)-driven tumors. Recent studies have identified new potential immunotherapeutic targets in CRC. However, due to the heterogeneous pathogenic basis of CRC, as well as other malignancies, a precise assessment of the therapeutic potential of specific immune targets requires a comprehensive understanding of their expression in relation to the status of critical mutations involved in tumor progression [14,15,16]. Considering SIGLEC9′s effect on immune responses and its potential role in tumorigenesis, it has recently been proposed as a target in various anti-cancer strategies [5,10,17]. Data regarding SIGLEC9 expression in CRC are currently inconclusive. To evaluate the potential of SIGLEC9 as a target for immunotherapy, further investigations into the molecular factors regulating SIGLEC9 expression are required.

The aim of this study was to investigate the expression of the SIGLEC9 protein in CRC tissues concerning the mutations in the KRAS, NRAS, BRAF, PIK3CA, and AKT genes, microsatellite instability (MSI) status, and the expression of cytokines, chemokines, and growth factors within the CRC TME.

## 2. Materials and Methods

### 2.1. Study Group Characteristics

This study involved 87 CRC tumors and 87 samples of corresponding surgical margins as the control group, all collected during CRC surgeries. The Research Ethics Committee provided approval for the study (PCN/0022/KB1/42/VI/14/16/18/19/20). The study group exclusively included tissues with histopathologically confirmed CRC diagnoses, and surgical margins free from pathological signs of CRC. The inclusion criteria required (1) patient consent, (2) an age above 18 years, and (3) histopathological confirmation of colorectal adenocarcinoma or tumor-free surgical margins. Patients failing to meet these criteria were excluded.

Clinicopathological features of the study group are detailed in Table 1.

### 2.2. SIGLEC9 Protein Concentration Measurment and Tissue Homogenization

The 87 CRC tumor and 87 margin tissues were homogenized following a previously established protocol [18]. Weighed samples were homogenized in a phosphate-buffered solution using a PRO 200 homogenizer (PRO Scientific Inc., Oxford, CT, USA) at 10,000 rpm, and then sonicated with an ultrasonic cell disrupter (UP100, Hilscher, Germany). Protein concentrations were measured using a pyrogallol-red reagent kit (Sentinel Diagnostics, Milano, Italy) in a Technicon RA-XTTM analyzer (Technicon Instruments Corporation, Mahopac, NY, USA) at 600 nm and 37 °C. The protein concentration of SIGLEC9 was determined using the Enzyme-linked Immunosorbent Assay (ELISA) method according to the assay protocol. Optimal sample dilutions for the SIGLEC9 ELISA kit were established in preliminary assay. The concentration of SIGLEC9 was measured in both study and control groups using ELISA kit SED922Hu (Cloud Clone, Wuhan, China) with sensitivity ~0.057 ng/mL. The results were recalculated to the corresponding total protein level and presented as ng/mL of protein.

### 2.3. KRAS, NRAS, BRAF, PIK3CA, and AKT Gene Mutations Assessment in CRC Tissues

To assess the mutation status of KRAS, NRAS, BRAF, PIK3CA, and AKT genes, we used real-time polymerase chain reaction (RT-PCR). Detailed methods have been described in a previous study [19]. Genomic DNA was extracted from fresh-frozen CRC tumors stored at −80 °C. From 69 tumor samples, DNA was isolated using an automated extractor with the commercial Mag-Bind Blood & Tissue DNA HDQ 96 Kit (M6399-00), following the manufacturer’s protocol and quantified by spectrophotometric analysis. According to the RT-PCR kit protocol, we adjusted the DNA concentration of each sample to 2 ng/μL to optimize the input DNA amount for further analysis. The CRC-RT48 Mutation Detection Panel for Real-Time PCR (EntroGen, Woodland Hills, CA, USA) was used to perform RT-PCR, targeting mutations in the examined genes, following the instruction manual protocol. The results were read and interpreted using the QuantStudio™ 5 Real-Time PCR System for Human Identification (Thermo Fisher Scientific, Waltham, MA, USA). The CRC-RT48 Mutation Detection Panel kit detects mutations in exons 2, 3, and 4 of KRAS and NRAS, exon 15 of BRAF, exons 9 and 20 of PIK3CA, and exon 4 of AKT1 (Table 2). This allowed us to determine the mutational status for KRAS 12/13, KRAS 117, KRAS 61, KRAS 146, KRAS 59, NRAS 12/13, NRAS 117, NRAS 61, NRAS 146, NRAS 59, BRAF 600, PIK3CA 542/545, PIK3CA 1047, and AKT1 E17K in the analyzed CRC tumors.

### 2.4. Microsatellite Instability Evaluation

For microsatellite instability (MSI) status analysis, we used formalin-fixed, paraffin-embedded CRC tissue blocks. MSI was determined via immunohistochemistry (IHC), examining MLH1, MSH2, MSH6, and PMS2 expression, as described in previous studies [20,21]. A formalin-fixed, paraffin-embedded (FFPE) tumor tissue block, sectioned at a thickness of 4 μm, was prepared and analyzed using a Dako Autostainer Link 48. Initially, the samples underwent deparaffinization and rehydration, followed by antigen retrieval, achieved by incubating the slides in EnVision Flex Target Retrieval Solution High pH (Dako, Carpinteria, CA, USA) at 95 °C for 20 min. Subsequently, the specimens were treated with Peroxidase-Blocked Reagent (Dako) and incubated with specific primary antibodies: the Mouse Monoclonal antibody MSH2 (clone G219-1129, Cell Marque, Rocklin, CA, USA) at a 1:400 dilution for 30 min; Mouse Monoclonal antibody MSH6 (clone 44, Cell Marque) at a 1:100 dilution for 45 min; Mouse Monoclonal antibody PMS2 (clone MRQ-28, Cell Marque) at a 1:50 dilution for 40 min; and Mouse Monoclonal antibody MLH1 (clone G168-728, Cell Marque) at a 1:100 dilution for 40 min. Afterward, EnVision FLEX HRP (Dako) was applied, followed by staining of the antigen–antibody complexes with 3,3′-diaminobenzidine (DAB). Finally, the sections were counterstained with hematoxylin, dehydrated, and mounted with coverslips for further evaluation. For the assessment of MSH2, MSH6, PMS2, and MLH1 expression, nuclear staining in invasive tumor cells was examined, with inflammatory and stromal cells serving as internal positive controls. Tumors displaying nuclear staining in at least 1% of invasive tumor cells were classified as having positive marker staining. The MSI status was confirmed if any of the following marker patterns were observed: loss of MLH1 and PMS2, loss of PMS2 alone, loss of MSH2 and MSH6, or loss of MSH6 alone.

### 2.5. Cytokine Screening Panel and Principal Component Analysis (PCA)

Cytokine, chemokine, and growth factor levels in 77 CRC homogenates were quantified using the Bio-Plex Pro Human Cytokine Screening Panel, 48-Plex (Bio-Rad Laboratories, Hercules, CA, USA), following the manufacturer’s protocol. Concentration values were adjusted by total protein levels and classified into groups based on Gene Ontology (GO) terms and KEGG annotations (Table 3) [22,23]. Principal component analysis (PCA) was performed on normalized expression data (decimal log-transformed). The covariance matrix was used to derive eigenvalues and eigenvectors, and three principal components were selected for further analysis. The component axes were rotated using the varimax method to better align positioning vectors and simplify the interpretation of factor loadings. We then assessed the correlations between the principal components and the SIGLEC9 protein expression. Due to the data distribution, we chose Spearman’s rank correlation for correlation analysis, with *p*-values < 0.05 considered significant. PCA was conducted using R Studio software 4.4.1. by applying the “factoextra” library.

### 2.6. Gene Set Enrichment Analysis (GSEA) for SIGLEC9 Gene Expression on CRC Data

The Gene Set Enrichment Analysis (GSEA) was conducted on the “FieldEffectCrc” dataset, specifically focusing on CRC samples from Cohort A [24] in R Studio 4.4.1. This cohort contained Salmon-generated transcript-level abundance estimates that were summarized to the gene level using tximport. The dataset included 834 human colorectal tissue samples across three phenotypes: tumor, normal adjacent-to-tumor, and healthy tissues [24]. The data were loaded using ExperimentHub and filtered to retain only the CRC samples (n = 311). Differential expression analysis was performed using the DESeq function from DESeq2 package, which normalized the count data to account for differences in sequencing depth between samples. Normalized counts were subsequently extracted using DESeq2. Samples were categorized based on the expression level of the gene ENSG00000129450 (SIGLEC9) by calculating its median expression across all samples. The median expression of the SIGLEC9 gene was determined to be 66.9149 normalized counts. Samples with expression values above the median were categorized as “high” (n = 156), while those with values below the median were categorized as “low” (n = 155). The results from the differential expression analysis are formatted and mapped to gene symbols using the org.Hs.eg.db database [25]. The ranked list of genes has been generated from the differential expression results, which will be used as input for the GSEA algorithm. Differential gene expression results were assigned to biological processes using the MSigDB (Molecular Signatures Database) hallmark gene sets collection [26,27]. Subsequently, GSEA was performed using the fgsea function from the fgsea package, with 10,000 permutations and additional filtering of gene sets based on size (minimum 15 genes and maximum 400 genes). The results were filtered to include only significant pathways with adjusted *p*-values (*p*-value < 0.05), and the pathways were ranked based on their normalized enrichment scores (NESs).

### 2.7. Statistical Analyses

The normality of the data was assessed using the Shapiro–Wilk test. The data were normalized through the use of a decimal logarithmic transformation. Non-normally distributed data were analyzed with the Mann–Whitney U test, while correlations between protein concentrations and TNM parameters were evaluated using Kendall’s Tau rank correlation. For examined protein expression and its association with mutation of examined genes, we used the Mann–Whitney U test. For examining other correlations, Spearman’s rank correlation was applied. Statistical significance was set at *p* < 0.05. The statistical analyses were performed in R Studio 4.4.1.

## 3. Results

### 3.1. SIGLEC9 Protein Expression in CRC Tissue and Surgical Margin Tissue

**Figure 1:**
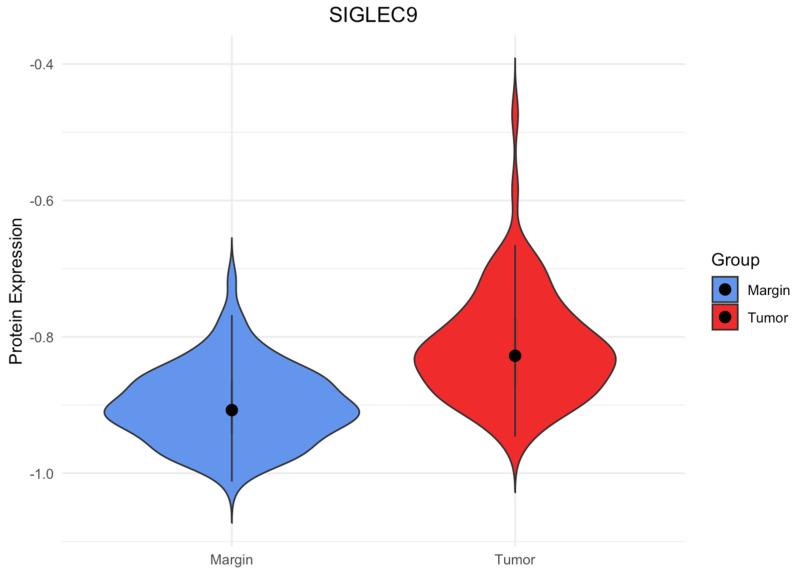
SIGLEC9 protein expression in CRC tissues and surgical margins. The data were normalized through the use of a decimal logarithmic transformation.

We measured SIGLEC9 protein concentrations in tissue homogenates of CRC and cancer-free surgical margins. We observed significantly elevated concentrations of the SIGLEC9 protein in CRC tissues compared to SIGLEC9 protein expression in the control group (*p* < 0.001). The levels of SIGLEC9 protein expression in the CRC tissue and surgical margin tissues are presented in Figure 1.

### 3.2. SIGLEC9 Protein Expression Based on Diverse Clinical and Pathomorphological Parameters

We aimed to investigate potential associations between SIGLEC9 protein expression and selected clinicopathological parameters. The analyzed parameters included the following: TNM scale parameters, tumor stage, tumor-infiltrating lymphocytes (TILs), grading, tumor localization (right-sided vs. left-sided tumors), and microsatellite instability (MSI) status.

**Figure 2:**
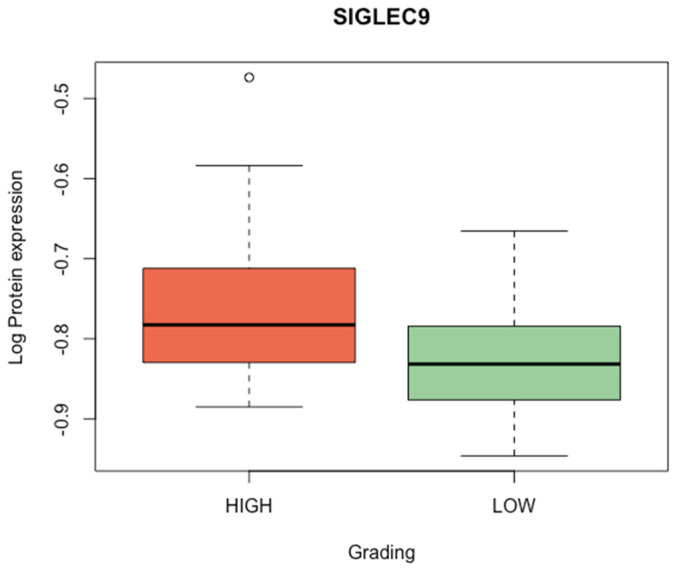
Box-plot illustrating SIGLEC9 protein expression in tumors with high grading versus low grading (Mann–Whitney U test, *p*-value < 0.001).

We observed a significantly higher expression of the SIGLEC9 protein in the group of tumors with high grading compared to the low grading tumors group (*p* < 0.001). Figure 2 presents SIGLEC9 protein expression based on tumor grading.

We did not observe significant differences in SIGLEC9 protein expression based on the primary tumor location (right-sided vs. left-sided tumors) (*p* > 0.05) or MSI status (*p* > 0.05). Moreover, SIGLEC9 expression did not significantly differ depending on the TNM scale parameters, tumor stage, or TILs (*p* > 0.05, Table 4).

### 3.3. Mutation Analysis of KRAS, NRAS, BRAF, PIK3CA, and AKT Genes with SIGLEC9 Expression

We investigated mutations in the KRAS, NRAS, BRAF, PIK3CA, and AKT genes using the RT-PCR method (details in Section 2.3) in a cohort of 69 CRC tumors.

Mutations in the KRAS gene were identified in 34.78% of tumors (n = 24), mutations in the NRAS gene in 15.94% (n = 11), and mutations in the BRAF gene in 7.25% of tumors (n = 5). Additionally, mutations in the PIK3CA and AKT genes were observed in 8.69% and 1.45% of tumors, respectively (n = 6 and n = 1). Among the identified KRAS mutations, the KRAS 12/13 mutation was the most prevalent, accounting for 66.67% (n = 16) of all KRAS mutations, while the KRAS 146 and KRAS 61 mutations were the least common, each occurring in 8.33% of cases (n = 2). Regarding NRAS mutations, the frequencies of NRAS 12/13 and NRAS 61 were 54.54% (n = 6) and 45.45% (n = 5), respectively. For the PIK3CA gene, the mutations PIK3CA 542/545 and PIK3CA 1047 were observed, respectively, in 83.33% (n = 5) and 16.67% (n = 1) of PIK3CA-mutant tumors. The frequencies of mutations in the KRAS, NRAS, BRAF, PIK3CA, and AKT genes in the studied cohort are presented in Table 5.

The data on mutations in the examined genes were used to verify whether SIGLEC9 protein expression differed based on the mutations of the examined genes. We did not observe significant differences in the SIGLEC9 protein expression levels based on the mutation status of KRAS, NRAS, BRAF, PIK3CA, or AKT genes (*p* > 0.05) (Mann–Whitney U test).

**Figure 3:**
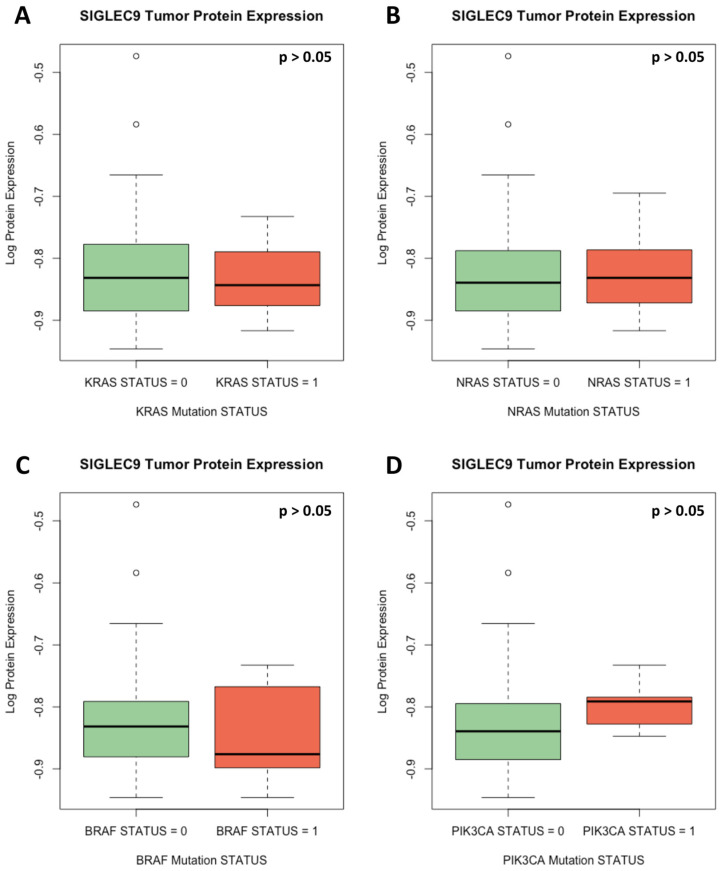
SIGLEC9 protein concentrations based on mutations in KRAS (**A**), NRAS (**B**), BRAF (**C**), and PIK3CA (**D**) genes. *p*—*p*-value from Mann–Whitney U test.

Figure 3 presents the SIGLEC9 protein expression based on the presence of positive or negative mutation status for the examined genes. The observed SIGLEC9 protein expression levels were independent of examined gene mutations.

### 3.4. Principal Component Analysis (PCA) for Groups of Cytokines Expressed in the CRC Tumor Microenvironment

To further explore the associations between SIGLEC9 protein expression and other cytokines, chemokines, and growth factors within the CRC TME, we employed principal component analysis (PCA) using data from the cytokine screening panel. The cytokines, chemokines, and growth factors were then categorized according to appropriate Gene Ontology (GO) terms and KEGG pathway annotations. The sets of molecules from the screening panel assigned to these terms are displayed in Table 3 of Section 2.

**Figure 4:**
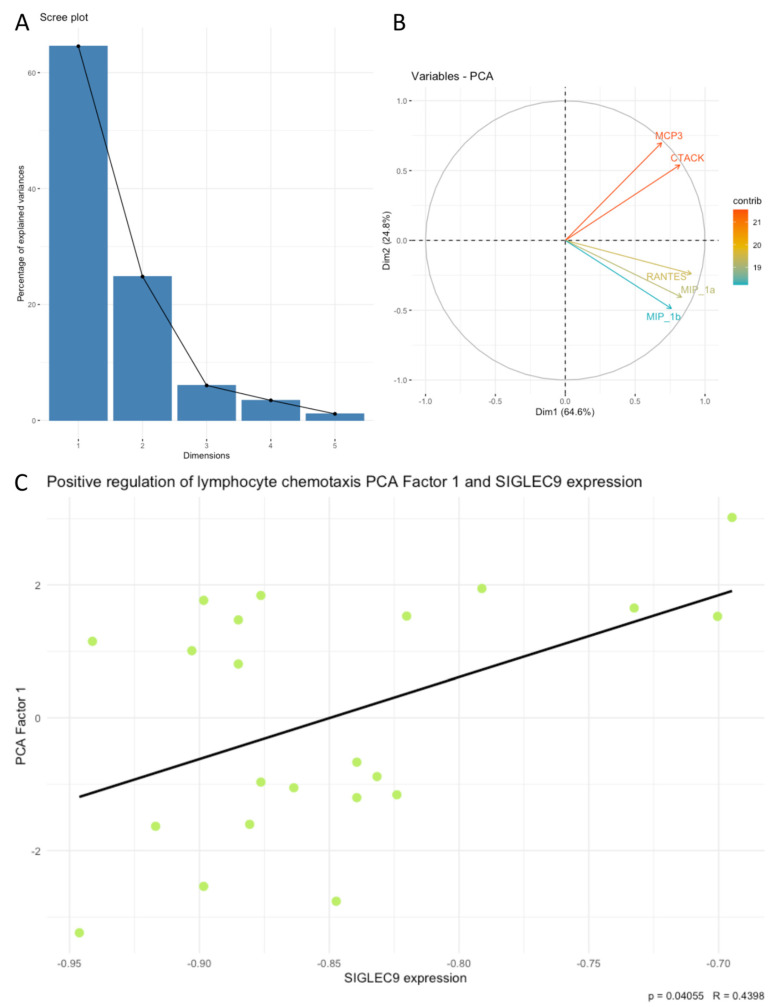
Scree plot (**A**) and biplot (**B**), and correlation plot (**C**) for positive regulation of lymphocyte chemotaxis set of cytokines and SIGLEC9 expression. The green dots on the plot represent data points from PCA used in the analysis of the correlation between PCA Factor 1 and the SIGLEC9 expression.

**Figure 5:**
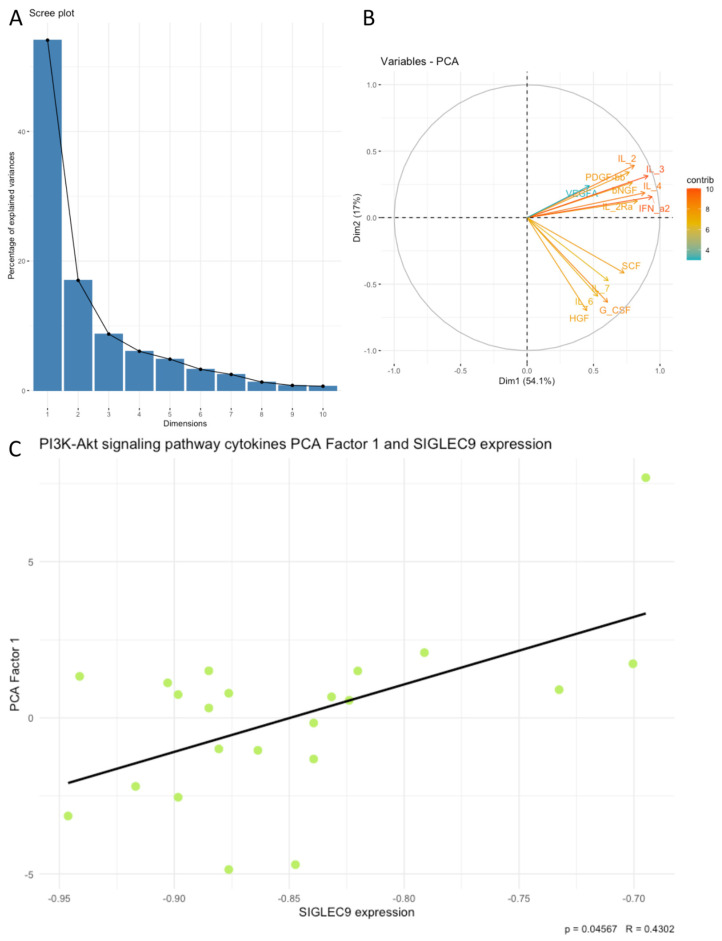
Scree plot (**A**) and biplot (**B**), and correlation plot (**C**) for PI3K-Akt signaling pathway set of cytokines and SIGLEC9 expression. The green dots on the plot represent data points from PCA used in the analysis of the correlation between PCA Factor 1 and the SIGLEC9 expression.

**Figure 6:**
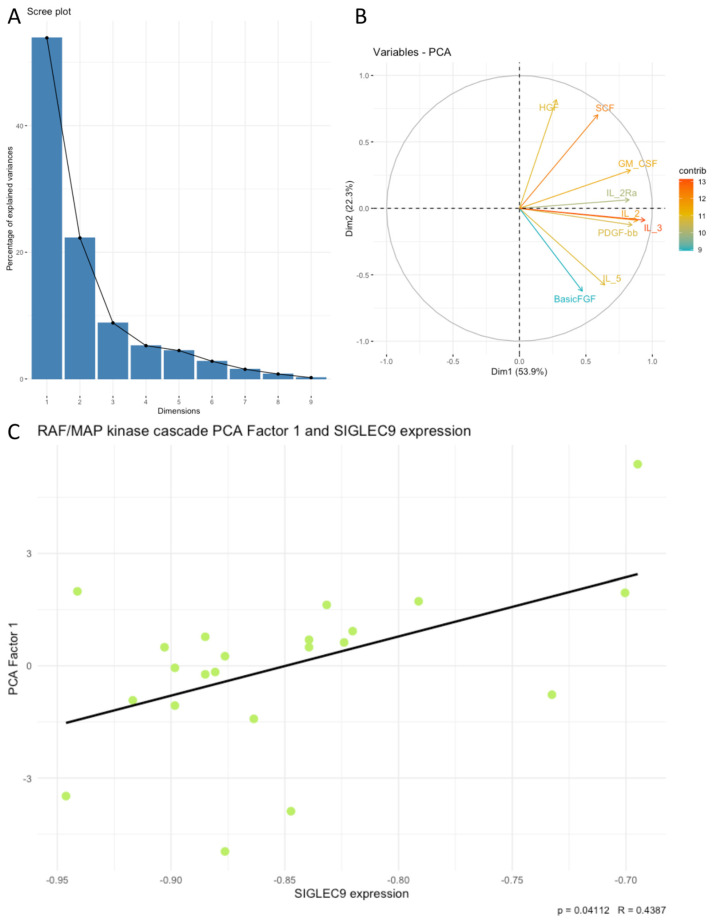
Scree plot (**A**) and biplot (**B**), and correlation plot (**C**) for RAF/MAP kinase cascade set of cytokines and SIGLEC9 expression. The green dots on the plot represent data points from PCA used in the analysis of the correlation between PCA Factor 1 and the SIGLEC9 expression.

**Figure 7:**
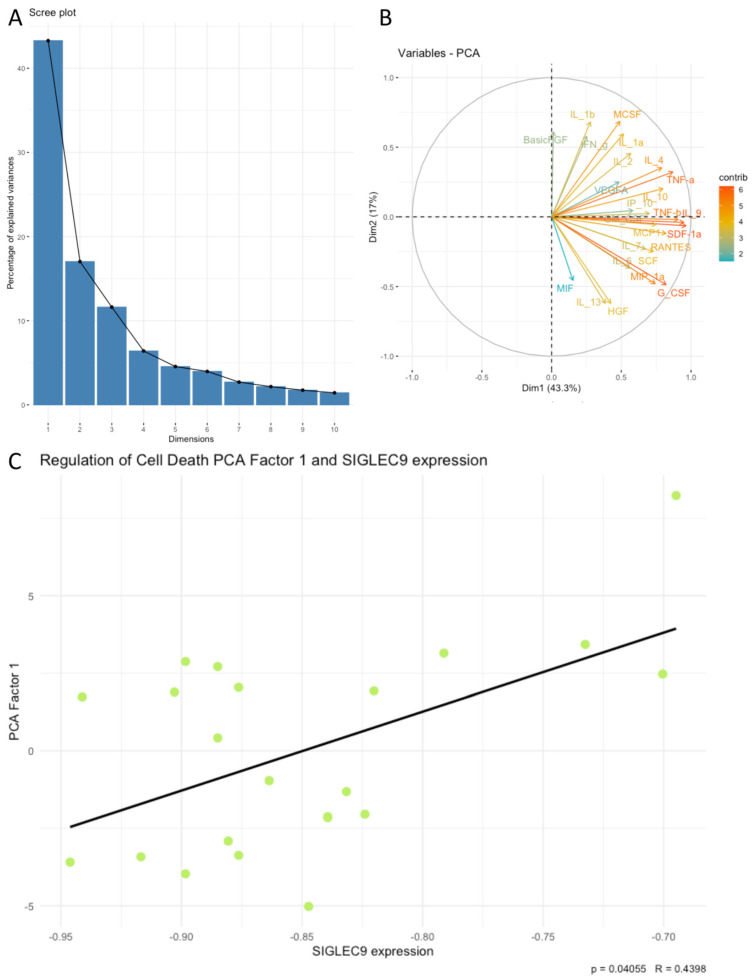
Scree plot (**A**) and biplot (**B**), and correlation plot (**C**) for regulation of cell death set of cytokines and SIGLEC9 expression. The green dots on the plot represent data points from PCA used in the analysis of the correlation between PCA Factor 1 and the SIGLEC9 expression.

**Figure 8:**
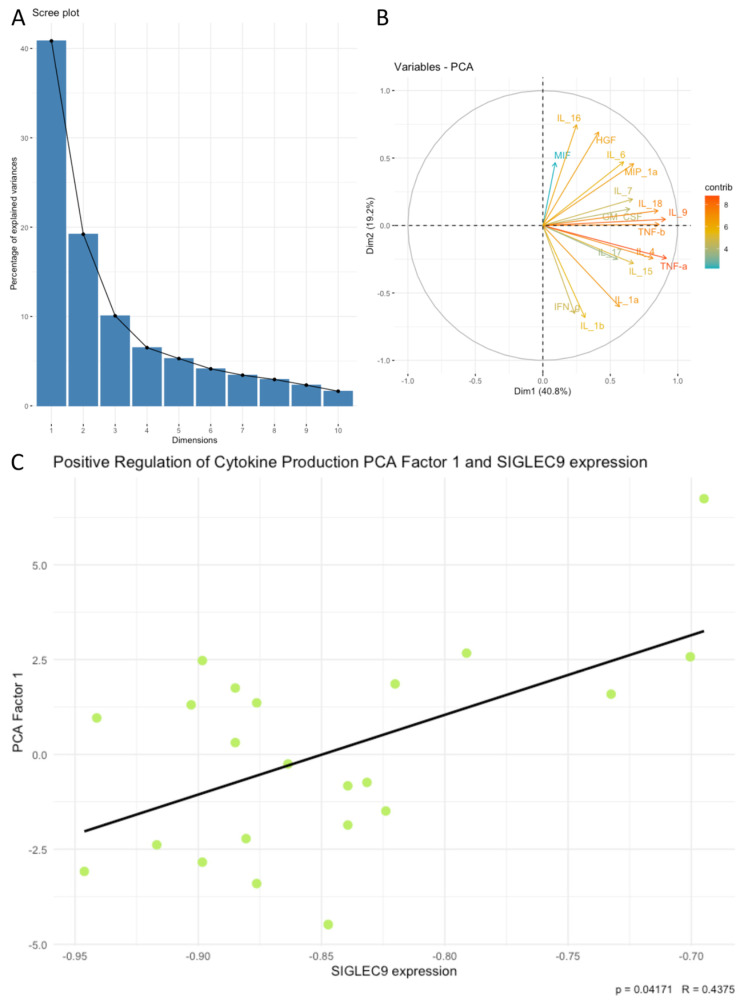
Scree plot (**A**) and biplot (**B**), and correlation plot (**C**) for positive regulation of cytokine production set of cytokines and SIGLEC9 expression. The green dots on the plot represent data points from PCA used in the analysis of the correlation between PCA Factor 1 and the SIGLEC9 expression.

Next, we examined associations between SIGLEC9 expression and the principal components (PCA factors) derived from the cytokines, chemokines, and growth factors. Our analysis revealed that SIGLEC9 expression was significantly associated with sets of examined cytokines related to positive regulation of lymphocyte chemotaxis, the PI3K-Akt signaling pathway, the RAF/MAP kinase cascade, regulation of cell death, and positive regulation of cytokine production. Notably, SIGLEC9 expression showed a positive correlation with the following PCA factors: positive regulation of lymphocyte chemotaxis Factor 1 (*p* < 0.05, R = 0.4398, Figure 4C), PI3K-Akt signaling pathway Factor 1 (*p* < 0.05, R = 0.4302, Figure 5C), RAF/MAP kinase cascade Factor 1 (*p* < 0.05, R = 0.4387, Figure 6C), regulation of cell death Factor 1 (*p* < 0.05, R = 0.4398, Figure 7C), and positive regulation of cytokine production Factor 1 (*p* < 0.05, R = 0.4375, Figure 8C). The eigenvalues and variance percentages for the three PCA factors related to positive regulation of lymphocyte chemotaxis, the PI3K-Akt signaling pathway, the RAF/MAP kinase cascade, regulation of cell death, and positive regulation of cytokine production are presented, respectively, in Table 6, Table 7, Table 8, Table 9 and Table 10. The coordinates for the variables in each set are detailed, respectively, in Table 11, Table 12, Table 13, Table 14 and Table 15. Scree plots (A) and biplots (B) for these cytokine groups included in the PCA are displayed, respectively, in Figure 4, Figure 5, Figure 6, Figure 7 and Figure 8. Among the cytokines examined, those with the most substantial influence on the first principal component in the PCA analysis of the ‘Positive regulation of lymphocyte chemotaxis’ were the following: RANTES (coordinates after varimax rotation = 0.9012), MIP-1a (coordinates after varimax rotation = 0.832), and CTACK (coordinates after varimax rotation = 0.819) (Table 7). The cytokines whose expression had the greatest impact on the first principal component in the conducted PCA analysis for the ‘PI3K-Akt signaling pathway’ were the following: IFN-a (coordinates after varimax rotation = 0.941), IL-3 (coordinates after varimax rotation = 0.910), IL-4 (coordinates after varimax rotation = 0.886), and IL-2 (coordinates after varimax rotation = 0.8072) (Table 9). In the PCA analysis of the ‘RAF/MAP kinase cascade’, the cytokines with the highest impact on the first principal component were the following: IL-3 (coordinates after varimax rotation = 0.945), IL-2 (coordinates after varimax rotation = 0.891), PDGF-bb (coordinates after varimax rotation = 0.848), and GM-CSF (coordinates after varimax rotation = 0.836) (Table 11). In the case of ‘Regulation of cell death ‘, it was the following: SDF-1a (coordinates after varimax rotation = 0.964), IL-2 (coordinates after varimax rotation = 0.948), GM-CSF (coordinates after varimax rotation = 0.908), RANTES (coordinates after varimax rotation = 0.870), IL-1a (coordinates after varimax rotation = 0.822), and IL-10 (coordinates after varimax rotation = 820) (Table 13), and in the case of ‘Positive regulation of cytokine production’, it was the following: TNF-a (coordinates after varimax rotation = 0.915), IL-9 (coordinates after varimax rotation = 0.908), TNF-b (coordinates after varimax rotation = 0.861), IL-18 (coordinates after varimax rotation = 0.853), and IL-4 (coordinates after varimax rotation = 0.816) (Table 15).

### 3.5. Gene Set Enrichment Analysis (GSEA) for High vs. Low Gene Expression of SIGLEC9 in CRC Tumors

For a deeper understanding of the interactions between the expression of SIGLEC9 with the molecular processes that occurred in the TME, we performed GSEA on the CRC tumor data. The GSEA results have also been used to validate our results from the PCA analysis on the cytokine expression data obtained from the cytokine screening panel.

**Figure 9:**
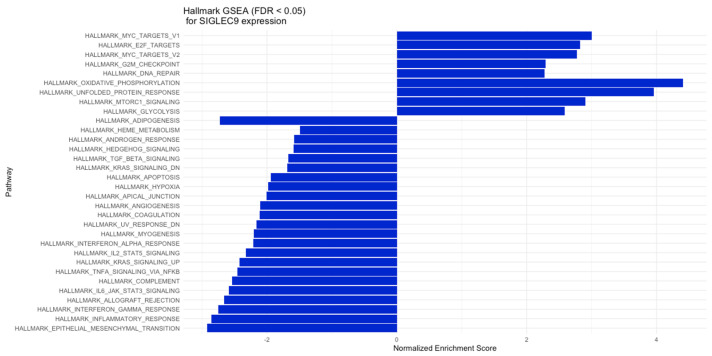
GSEA results for high versus low SIGLEC9 expression, where the *X*-axis represents the NES (normalized enrichment score) and the *Y*-axis displays various biological processes and signaling pathways from the hallmark gene sets collection from the MSigDB (Molecular Signatures Database).

The conducted GSEA revealed that hallmark gene sets with positive normalized enrichment scores (NESs) in the high SIGLEC9 expression group were linked to MYC targets, E2F targets, the G2M checkpoint, DNA repair, oxidative phosphorylation, the unfolded protein response, and MTORC1 signaling. Conversely, hallmark gene sets with negative NESs for SIGLEC9 expression were predominantly associated with inflammation, cytokine signaling, apoptosis, hypoxia, and angiogenesis. These processes included TGF-beta signaling, Interferon-alpha response, IL-2/STAT5 signaling, Interferon-gamma response, inflammatory response, complement-associated genes, and epithelial–mesenchymal transition, among others. Furthermore, in the low SIGLEC9 expression group, enriched hallmark gene sets included Hedgehog signaling and KRAS signaling regulation (Figure 9).

## 4. Discussion

In our study, we observed significantly elevated levels of the SIGLEC9 protein in CRC tissues compared to non-cancerous surgical margin tissues (*p* < 0.001). The distribution of patients across different stages of clinical staging in the study group is relatively even, with the highest frequency of tumors in stages II and III (26.43% and 42.53%, respectively), which seems to reflect population-level frequencies. Our findings differ from those of Wu et al. [3], which may be due to the limitation of our study, such as the small sample size (n = 87). However, the other literature data examining SIGLEC9 expression in CRC can be found. As mentioned earlier, sialic acids and SIGLECs likely play an important role in maintaining immunological homeostasis in the colon, and alterations in SIGLEC9-related signaling could lead to cancerogenesis. Still, very few works exist tackling the issue of SIGLEC9′s role in the colon and CRC. Miyazaki et al. uncovered that SIGLEC7 and SIGLEC9 are expressed on resident macrophages in colonic lamina propria with high CD68/CD163 expression, but also to a lesser extent on CD8+ T lymphocytes and other cells, mostly granulocytes. Nonmalignant colonic epithelial cells expressed disialyl Lewisa, SIGLEC7/9 ligand, and sialyl 6-sulfo Lewisx, which binds to SIGLEC7. Carcinogenesis led to the transition of the mentioned ligands into sialyl Lewisa and sialyl Lewisx, with no binding ability to SIGLEC7/9. Normal glycans on epithelial cells in the colon exerted immunosuppressive activity, inhibiting the cyclooxygenase-2 expression and PGE2 synthesis in macrophages, playing an immunomodulating role in colonic mucosa [1]. Thus, carcinogenesis could abolish this immunological homeostasis, increasing inflammatory responses. In a study conducted by Chang et al., SIGLEC9 expression on macrophages in 13 samples collected from colon cancer (CC) patients was significantly higher in cancer tissues than in peritumor tissues. Elevated SIGLEC9+ TAMs in CC were associated with an immunosuppressive environment, as well as a lower fraction and altered function of CD8 + T cells. Moreover, in such macrophages, there was an upregulation in immune checkpoint genes including PD-1 (PDCD1), CTLA-4, and TIM3 (HAVCR2). The authors revealed a positive correlation between SIGLEC9 expression in TAMs and immune infiltrations. Such infiltrations were especially abundant in immunosuppressive cells, including TAMs, Tregs, and neutrophils, as well as a higher fraction of M2 macrophages. Authors suggested that SIGLEC9 TAMs may influence Treg and CD8+T cell function through CTLA-4, leading to their dysfunction. SIGLEC9 + TAM infiltrations also corresponded to the upregulation in the VEGF signaling, suggesting that SIGLEC9-associated pathways may influence angiogenesis [2]. Notably, higher infiltrations of SIGLEC9 + TAMs related to poorer patient survival (*p* < 0.001), but also a better response to 6-month adjuvant chemotherapy, which manifested with significantly longer disease-free survival (DFS) and overall survival (OS). The role of SIGLEC9 in CRC was studied by Egan et al. [11]. The authors revealed that the elevated expression of α2,3 and α2,6 sialic acid in mesenchymal stromal cells (MSCs) may create an immunosuppressive environment, inhibiting the proliferation of CD4+ and CD8+ T cells in tumors. Interestingly, tumor-conditioned stromal cells exhibited significantly higher expression of α2,6 sialic acid than CC epithelial cells. Treatment with sialyltransferase inhibitor (SI) could partially reverse the inhibitory effect of tumor-conditioned MSCs on CD4 and CD8 cell proliferation. SIGLEC-E, an SIGLEC7/9 analog, was overexpressed in CD4+ and CD8+ tumor-infiltrating T cells, and its levels were also dependent on sialyltransferase activity. Moreover, SI pretreatment increased the number of CD8+granzyme B+ T cells. In addition, α2,3-specific sialyltransferases were overexpressed in the stromal compartment of human CRC tissues compared to the epithelial fraction, while the expression of ST6GAL1 and ST6GAL2 (α2,6 linkage-specific sialyltransferases) was increased in human CRC cancer-associated fibroblasts (CAFs) compared to the controls. SIGLEC9, out of the whole SIGLEC family, had the strongest positive association with fibroblast activation protein. Also, CAFs presented significantly higher levels of SIGLEC9 than normal fibroblasts (NAFs). CAFs derived from CRC patients presented stronger suppression of CD4+ and CD8+ T cell proliferation than NAFs. Again, SI pretreatment inhibited the expression of the SIGLEC9 ligand on CAFs. These findings indicate that sialic acids in stromal cells play an important role in immunomodulation in the tumor microenvironment (TME) in CRC [11].

The expression of SIGLEC9 has been studied in various cancers, with divergent results regarding its upregulation or downregulation in cancer tissues. Meril et al. confirmed overexpression of SIGLEC9 ligands in various neoplastic cell lines, including melanoma, leukemia, or prostate cancer cells [9]. Sia-SAMPs (SIGLEC7 and SIGLEC9 ligands) were also upregulated in lung carcinomas compared to healthy tissue [10]. Sialic acid expression was increased in pancreatic ductal adenocarcinoma (PDAC) as well, and α2,3 sialic acids are likely the main targets for SIGLEC9 in PDAC [28]. As shown by Eisenberg et al., IFNγ is able to induce α2,6-hypersialylation and thus augment the number of SIGLEC9 ligands in cancer cell lines in vitro. Additionally, SIGLEC9+ T cells had impaired effector function, while SIGLEC9 knockdown could restore T cell activity. SIGLEC9 overexpression on T cells reduced the secretion of INFy and TNFa against melanoma. CMP-sialic acid synthase (CMAS) knockdown in PDAC revealed that sialic acids in tumor cells induce an immunosuppressive environment by increasing IL-10 secretion and CD206 expression. They could also alter TAM differentiation in an SIGLEC7/9-dependent manner [5]. Rodriguez et al. demonstrated that α2,3 sialic acids may play a role in monocyte transformation into macrophages with immunosuppressive properties by increasing IL-10, IL-6, and PD-L1 expression. They also caused the phosphorylation of SIGLEC9, but not SIGLEC7. A similar process occurred in the case of already differentiated macrophages, meaning that α2,3 sialic acids could induce TAM differentiation via SIGLEC9 to create an immunosuppressive environment in PDAC [28]. Different tumor types vary regarding SIGLECs expression and function. TCGA and GTEx databases revealed that SIGLEC9 is strongly expressed in numerous tumors, including breast cancer, gliomas, head and neck squamous cell carcinoma (HNSC), kidney tumors, stomach adenocarcinoma (STAD), thyroid carcinoma (THCA), cervical cancer, esophageal carcinoma (ESCA), ovarian cancer, skin cutaneous melanoma (SKCM), testicular germ cell tumors (TGCT), or uterine cancer [3]. SIGLEC9 was also upregulated in TILs in CRC according to another study [10]. At the same time, its decreased expression was found in colon adenocarcinoma (COAD), liver hepatocellular carcinoma (LIHC), lung cancer, and adrenocortical carcinoma (ACC), compared to the control samples [3]. SIGLECs expression in pancreatic ductal adenocarcinoma (PDAC) can be found mainly in myeloid cells [28]. In primary triple-negative (TN) breast cancer, SIGLEC7/9 expression is higher compared to estrogen receptor-positive breast cancer and SIGLEC9 was present on almost all (>90%) of the CD11b+ cells, being mostly absent on CD3+ or CD56+ cells with high SIGLEC7 expression. Interestingly, immune infiltrations in TN were more prominent than in estrogen receptor-positive breast cancer [29]. In NSCLC, SIGLEC9 was coexpressed with CD3 and Sig9+CD8+ TILs exhibited strong PD1 expression and seemed to be a subpopulation of PD1-high CD8 TILs. In addition, Sig9+ CD8 cells reacted differently than Sig9- cells upon activation. Sig9+ cells exhibited higher surface levels of CD25, CD5, CD69, and SIGLEC5, but they were also rich in inhibitory receptors and produced fewer cytokines, such as IFN-γ and TNF-α. On the other hand, they could be restimulated much easier to secrete various cytokines at higher levels. Moreover, such cells expressed different sets of chemokine receptors, which included CXCR3, CXCR5, CCR4, CCR6, and CX3CR1. Such results highlight the importance of SIGLEC9 in immune responses in cancer, pointing out that its altered expression may abrogate the activity of immune cells in certain cancer types [10].

In various malignancies, SIGLEC9 was expressed differently depending on the clinical stage of a patient or a molecular subtype of the cancer. The prognostic potential of SIGLEC9 depended on the cancer type as well [3]. In skin cutaneous melanoma (SKCM), high SIGLEC9 expression correlated significantly with longer OS, progression-free interval, and disease-specific survival, while in hepatocellular carcinoma (HCC), SIGLEC9 overexpression on NK cells correlated with a worse prognosis [4,17]. SIGLEC9 upregulation was related to poorer overall survival (OS) and 5-year OS in lung cancer, esophageal carcinoma, and low-grade gliomas (LGGs), while the opposite results were obtained for adenoid cystic carcinoma (ACC). Better outcomes were observed for platinum-free interval (PFI) in SIGLEC9-high ACC patients, but higher SIGLEC9 expression was related to worse PFI outcomes in glioblastoma, prostate adenocarcinoma, and LGG [3]. In brain lower-grade glioma (LGG), SIGLEC9 was expressed most abundantly in grade IV WHO tumors, and high SIGLEC9 levels were associated with poorer prognosis in primary WHO II and recurrent WHO III LGG [3]. In CC, SIGLEC9+ TAM infiltrations correlated positively with elevated rates of lymph node and distant metastasis. There was a significant positive correlation with a more severe TNM stage (*p* < 0.001), while SIGLEC9+ TAM concentrations acted as an independent prognostic factor for OS in CC [2]. This means that SIGLEC9 may perform different functions depending on the cancer type, influencing the clinical characteristics and survival of the patients.

In our study, we did not observe significant correlations between the expression of the SIGLEC9 protein and the parameters of the TNM scale or the cancer stage. The expression of SIGLEC9 was elevated regardless of the T, N, M parameters and staging when compared to the surgical margin tissues. However, we did observe a significantly increased expression of the SIGLEC9 protein in the group of high-grade CRC tumors compared to the low-grade tumors group. Given that high-grade CRC tumors are generally characterized by increased cellular proliferation and often exhibit a more aggressive phenotype, the observed upregulation of SIGLEC9 protein expression—an established immune checkpoint that suppresses immune responses—suggests that SIGLEC9 could be involved in immune surveillance evasion more in high-grade tumors than in low-grade CRC tumors. This potential for immune evasion in high-grade tumors is particularly concerning, as such tumors are often associated with a poorer prognosis. The prognostic significance of SIGLEC9 in the clinical course of CRC warrants further investigation, particularly in light of the negative prognostic implications linked to high-grade tumors. However, it is important to note that this study does not elucidate the specific mechanisms responsible for the increased expression of SIGLEC9 in high-grade CRC tumors. Understanding these mechanisms is critical for assessing the therapeutic potential of targeting SIGLEC9 and other immune checkpoints. Future research should focus on exploring the role of SIGLEC9 in CRC more comprehensively, especially considering its implications for developing targeted immunotherapies. Given the complexity of the TME and the interplay between various immune markers, elucidating the relationship between SIGLEC9 expression and tumor characteristics could significantly enhance our understanding of its prognostic value and potential as a therapeutic target.

The KRAS and NRAS genes, variants of the RAS family, play a crucial role in CRC due to both their mutation frequency and their regulatory influence on pathways controlling cell proliferation, motility, angiogenesis, and survival. Additionally, oncogenic KRAS drives the production of inflammatory cytokines and chemokines, significantly impacting the immune TME through downstream signaling pathways [30,31,32]. Mutations in the KRAS gene are found in approximately 30–50% of CRC cases, establishing it as the most frequently altered oncogene in this malignancy [33,34]. In contrast, mutations in the NRAS gene are comparatively rare, detected in around 3–10% of CRC cases [35,36]. KRAS mutations predominantly occur in exon 2, particularly at codons 12 and 13, while mutations in exons 3 and 4 are less frequent [37,38]. For instance, a study examining 408 CRC tumors reported that 33.1% (n = 135) of tumors exhibited KRAS exon 2 mutations, while NRAS mutations in exons 2 and 3 were identified in 2.4% of cases (n = 10) [39]. Another study found comparable KRAS mutation frequencies, with over 90% of KRAS mutations occurring in exon 2 (68.9% at codon 12 and 24.4% at codon 13). NRAS mutations appeared in 8.8% of cases, with frequencies of 57.1%, 28.6%, and 14.3% in exons 2, 3, and 4, respectively [40]. In our cohort of 69 patients selected for mutation analysis, we observed a 66.67% frequency of KRAS exon 2 mutations, corresponding to KRAS 12/13 mutations, aligning with findings from other studies. In exons 3 and 4, mutation frequencies were 8.33% each, which were higher than the reported rates of 2.7% and 3.7% in other studies. NRAS mutations in our cohort appeared in 18% of cases, which was a higher rate than those reported in previous studies (2.5% and 8.8% for NRAS mutations), with 54.54% of mutations in exon 2 and 45.45% in exon 3. These discrepancies may be attributed to variations in patient demographics, including age, gender distribution, disease stage, and ethnic backgrounds. In this study, we analyzed mutations in BRAF exon 15, specifically V600E, V600E2, V600D, and V600K (Table 2), finding a mutation frequency of approximately 7% among all examined tumors, consistent with findings in other studies [41,42]. PIK3CA mutations were observed in 10–30% of CRC cases, while AKT1 mutations remained relatively rare at around 1% [43,44]. In our cohort, PIK3CA mutations in exons 9 and 20 had a frequency of about 8.69%, slightly lower than reported in other studies, while AKT1 mutation frequencies aligned closely with other reports. In our study, we did not observe significant differences in SIGLEC9 protein expression depending on mutations in the KRAS, NRAS, BRAF, PIK3CA, and AKT genes. To the best of our knowledge, no studies have yet addressed the associations between SIGLEC9 expression and mutations in the KRAS, NRAS, BRAF, PIK3CA, and AKT genes. The results presented here suggest that SIGLEC9 expression is regulated independently of these gene mutations. This could be due to mutations in other genes, such as TP53, which we did not analyze in our study, or increased SIGLEC9 expression may potentially occur at the very early stages of CRC pathogenesis during the induction of primary mutations. Considering SIGLEC9 as a potential molecular target, our results suggest that the clinical effects of potential therapies based on inhibiting SIGLEC9 expression may be consistent, regardless of the mutation status of the KRAS, NRAS, BRAF, PIK3CA, and AKT genes in CRC patients. The frequency of KRAS, NRAS, BRAF, PIK3CA, and AKT mutations in CRC observed in our cohort of 69 tumors was mostly consistent with the mutation rates reported in other studies. However, the number of tumors with mutations in specific genes was relatively small in our analysis, which certainly poses a limitation. Therefore, our results should primarily be considered as preliminary findings. Due to the prevalence of these mutations in CRC, validation of our findings in studies with larger patient cohorts is required. To the best of our knowledge, no studies have yet explored the associations between SIGLEC9 expression and mutations in the KRAS, NRAS, BRAF, PIK3CA, and AKT genes.

Similarly, we did not observe differences in SIGLEC9 protein expression based on MSI status. However, associations between SIGLEC9 expression and MSI status have been previously noted in other studies. MSI status has been negatively associated with SIGLEC9 expression in head–neck squamous cell carcinoma (HNSC), glioma, lung cancer, pancreatic adenocarcinoma (PAAD), SKCM, and tenosynovial giant cell tumors (TGCTs), but positively in CRC. Among Mismatch Repair (MMR) genes, MLH1, MSH2, MSH6, PMS2, and EpCAM were significantly associated with SIGLEC9 levels, with EpCAM showing a mostly negative correlation. Interestingly, SIGLEC9 expression was also related to DNA methylation in various cancer types, and the protein levels were associated positively with DNA methyltransferase1 (DNMT1), DNA methyltransferase2 (DNMT2), and DNA methyltransferase3 (DNMT3) expression [3]. MSI status was analyzed in 73 tumors in our cohort. Together with the low frequency of positive MSI status in our group (17.81%), although consistent with the literature data, this represents a limitation of our study. Therefore, our observation should be considered as a preliminary finding and verified in studies with a larger sample size.

Our analysis demonstrated that SIGLEC9 expression exhibited a positive significant correlation with the positive regulation of lymphocyte chemotaxis (*p* < 0.05, R = 0.4398), PI3K-Akt signaling pathway (*p* < 0.05, R = 0.4302), RAF/MAP kinase cascade (*p* < 0.05, R = 0.4387), regulation of cell death (*p* < 0.05, R = 0.4398), and positive regulation of cytokine production (*p* < 0.05, R = 0.4375). The GSEA results are primarily consistent with our PCA results; where the most enriched processes in high or low SIGLEC9 expression were immune processes, we have revealed them to be associated with SIGLEC9 expression in PCA on our cytokine data. Cytokines with a significant impact on principal component Factor 1 in the context of the regulation of cell death processes include SDF-1α (stromal cell-derived factor 1 alpha), IL-1α, and IL-10. SDF-1α is a chemokine that plays an essential role in recruiting inflammatory cells, including T lymphocytes and monocytes, to the TME. In the context of CRC, SDF-1α overexpression may facilitate the migration of immune cells, potentially fostering immunosuppression through the recruitment of suppressive cells such as myeloid-derived suppressor cells (MDSCs) and regulatory T cells (Tregs) [45,46,47]. IL-10 is a key immunosuppressive factor that attenuates the immune response by inhibiting T cell and macrophage activity. Within the TME, it acts as a primary regulator of immunosuppression, thereby supporting tumor survival [48]. IL-2 plays a dual role, stimulating the proliferation of effector T lymphocytes that can fight the tumor while also being critical for the development and function of Tregs. Thus, in the TME, IL-2 may both support the immune response and promote immunosuppression by promoting Treg expansion [49,50]. The potential regulatory interactions appear to be partially elucidated by the analysis of the GSEA results. An increase in the SIGLEC9 levels may lead to cellular stress and activation of the unfolded protein response (UPR), a common adaptive mechanism in highly proliferative tumors. It can induce the expression of cytokines such as IL-10, which promote immunosuppression, supporting cancer cell evasion of the immune surveillance. The enrichment of IL-6/JAK-STAT3 and IL-2/STAT5 signaling pathways in low SIGLEC9 expression suggests that lower SIGLEC9 levels may activate pro-inflammatory pathways that support an anti-tumor immune response by enhancing cytotoxic T cell activity. Conversely, high SIGLEC9 expression may antagonistically suppress these pathways, promoting more immunosuppressive TME characteristics. SIGLEC9 expression, associated with the activation of TGF-beta and Interferon responses, may support inflammation and promote the recruitment of immune cells, including macrophages and dendritic cells. These processes counteract immunosuppression and can enhance the anti-tumor response in cases of lower SIGLEC9 expression. Enrichment in MTORC1, MYC, and UPR in the GSEA results in the high SIGLEC9 expression tumor group may promote proliferation and adaptive survival mechanisms, creating an environment conducive to cytokine production, such as IL-10, SDF-1α, and IL-2. These cytokines, in turn, support the recruitment of immunosuppressive cells and may establish a TME favorable to tumor survival.

In turn, for the observed association between SIGLEC9 expression and cytokines, chemokines, and growth factors involved in the regulation of cell death processes, the most significant cytokines, analyzed in this context, were TNF-α, IL-9, and IL-4. TNF-α is a strongly pro-inflammatory cytokine that activates various immune cells, such as macrophages, neutrophils, and lymphocytes. It may induce an inflammatory cascade within the TME, promoting angiogenesis and metastasis. In CRC, TNF-α may contribute to establishing a protumorigenic inflammatory environment that facilitates tumor progression and resistance to anti-cancer therapies [51,52,53]. IL-9 is often associated with the function of type 2 helper T cells (Th2) and mast cells. In cancer, it may promote immunosuppressive responses and support tumor growth by stimulating cancer cell proliferation and protecting against apoptosis [54,55]. IL-4 is known to influence the development of Th2 cells and promote humoral responses, which may support immunosuppression. In the TME, IL-4 may support the proliferation of Tregs and M2 macrophages, which encourage cancer progression and immunosuppression [56,57,58]. Both IL-9 and IL-4 are components of the tumor’s immunosuppressive microenvironment, aiding cancer cells in evading immune responses. Similarly, to what was previously discussed, the presented GSEA analysis results may elucidate observed associations between SIGLEC9 and cytokines IL-4, IL-9, and TNF-α, as well as their potential collaborative roles in creating immunosuppressive characteristics of the TME. High SIGLEC9 expression is associated with the enrichment of genes involved in pathways such as MYC, E2F, G2M checkpoint, mTORC1, and the response to misfolded proteins. These processes are critical for the rapid proliferation and survival of cancer cells in the TME. This activation may promote tumor development by supporting cellular processes essential for its survival and growth. In the context of the associations with IL-4, IL-9, and TNF-α, high SIGLEC9 expression may have a suppressive effect on the immune system, helping the tumor evade elimination by immune cells. The enrichment of proliferative and metabolic pathways, coupled with the suppression of genes linked to angiogenesis and hypoxia, suggests that SIGLEC9 may support cancer cell adaptation to challenging tumor conditions without engaging processes like angiogenesis. Suppression of genes related to apoptosis and inflammation indicates that SIGLEC9 may support cancer cell survival. The GSEA results for SIGLEC9 suggest that it is involved in processes regulated by MYC, mTOR, and E2F, pathways commonly engaged in the control of cancer cell growth and survival. These pathways may also indirectly regulate IL-4, IL-9, and TNF-α, as these cytokines frequently interact with metabolic and proliferative pathways within the tumor microenvironment.

The associations we have revealed in the PCA in the case of associations between the cytokines associated with PI3K-Akt signaling have been supported by current evidence. SIGLEC9 triggered a signal that led to the degradation of focal adhesion kinase (FAK), associated in a great manner with PI3K-Akt signaling, in tumor cells through an interaction between SIGLEC9 on immune cells and its coreceptors on tumor cells via sialylglycoconjugates, leading to modulation of tumor cell adhesion kinetics. This relationship was demonstrated by co-culturing AS cells with U937 with high or low levels of SIGLEC9 and examining the activation of representative proteins. In AS cells co-cultured with U937 SIGLEC9-high, the total number of FAK bands decreased dramatically, resulting in all phosphorylation site-specific FAK bands showing a marked reduction in band intensity. In contrast, minimal changes in FAK and/or phosphorylated FAK were observed in AS cells co-cultured with U937 SIGLEC9-low cells [59]. Von Gunten et al. showed that SIGLEC9 plays a key role through a caspase-dependent pathway in the absence of cytokines in triggering apoptotic neutrophil death [60]. In addition, they showed that this cytotoxicity can be augmented by pro-inflammatory cytokines, including granulocyte-macrophage colony-stimulating factor (GM-CSF), Interferon (IFN)-α, and IFN-γ. No ROS was detected in ROS collectors or neutrophils, confirming that ROS-induced neutrophil death is related to caspase-dependent and -independent mechanisms. There is also some evidence that SIGLEC9 binding to natural anti-SIGLEC9 antibodies occurs on neutrophils after treatment with intravenous immunoglobulin (IVIg) preparations. This effect was observed to accelerate dramatically in a cytokine-rich microenvironment. Parallel to the reported effects of SIGLEC9 ligation by specific antibodies, neutrophil death mediated by IVIg also involves ROS, in a caspase-dependent and caspase-independent manner. To date, the mechanisms leading to caspase activation in SIGLEC9-induced neutrophil death remain poorly understood [60,61]. Regarding the association between SIGLEC9 expression and the positive regulation of cytokine production, there is also some evidence that sheds light on a better understanding of the observed association. SIGLEC9 affects the expression of various cytokines. The available reports demonstrate its anti-inflammatory properties and potential in the treatment of autoinflammatory diseases. These properties could also have important implications for cancer therapies. SIGLEC9 likely mediates IL-4-dependent activation of the PI-3K/Akt pathway. Neither SIGLEC9 nor IL-4 alone are able to induce strong Akt phosphorylation, but the levels of phosphorylated Akt significantly rise after IL-4 stimulation and SIGLEC9 treatment [62]. Higuchi et al. uncovered that SIGLEC9 knockdown augmented CCR7 expression and reduced IL-4 secretion upon stimulation with LPS plus IFN-γ. The authors suggested SIGLEC9 as the main modulator of inner immunity in blood monocytes and macrophages among SIGLEC family proteins. SIGLEC9 expression was also enhanced approximately two-fold after M-CSF and GM-CSF stimulation [63]. In another study, the treatment of bone marrow macrophages (BMMs) with MCP-1/sSIGLEC9 (soluble SIGLEC9) resulted in a stronger tendency for Il-10, VEGF, and Igf secretion [64]. Moreover, pretreatment of macrophages with human anti-SIGLEC9 Fab fragment (hS9-Fab03) constructed by Chu and colleagues led to decreased LPS-induced TNF-α, IL-6, IL-1β, IL-8, and IFN-β production in human peripheral blood mononuclear cell (PBMC)-derived macrophages, while slightly increasing IL-10 levels [65]. As revealed by Ge et al., in patients with acute exacerbation of chronic pulmonary disease (AECOPD), an increase in the expression of SIGLEC9 in the peripheral blood neutrophils augmented inflammatory markers including CRP, IL-6, IL-8, and TNF-α expression [66]. Soluble SIGLEC9 was also studied as a potential therapeutic strategy in intestinal inflammation. sSIGLEC9 decreased IL-8 and TNF-α gene expression in stimulated COLO 205 and RAW 264.7 cells, alleviating colitis in mouse models [67]. The analysis of directional associations between SIGLEC9 expression and immune processes, as examined through PCA, alongside a comparison with GSEA outcomes for SIGLEC9 expression, faces certain limitations. Specifically, while investigating these processes in PCA, we included only a limited subset of cytokines, chemokines, and growth factors related to the respective processes, despite their actual number being substantially higher. The broad scope of GSEA contributes to greater accuracy in the results obtained. Given these and other limitations in the research methods used, we regard our findings as preliminary. Nevertheless, both the outcomes of our experimental investigations and statistical analyses, as well as GSEA findings, suggest a link between SIGLEC9 protein expression and the examined immune processes in CRC. Considering that the regulatory mechanism between SIGLEC9 and the expression of the cytokines associated with the immunosuppressive effect in the TME remains unknown, several possible scenarios could explain the observed correlations. In this context, the associations between SIGLEC9 and these cytokines might be indirect, suggesting that the expression of both SIGLEC9 and these cytokines could be jointly regulated by other factors. Should the expression of SIGLEC9 and these cytokines arise from shared regulatory pathways, their inhibition may require a more complex therapeutic approach. Targeting pathways of common regulation or overarching factors, such as specific microRNAs, shared transcriptional factors, or hypoxia modulators, could potentially exert a broader impact on the tumor’s immunosuppressive microenvironment. Blocking these shared elements may weaken tumor-induced immunosuppression and restore the immune system’s ability to target cancer cells. Additional studies using cell cultures or animal models, which were beyond the scope of this research, are necessary to clarify potential regulatory interactions between SIGLEC9 and the expression of these cytokines. Further research is essential to fully understand the impact of altered SIGLEC9 expression on the immune properties of the TME.

## 5. Conclusions

The expression of SIGLEC9 was significantly elevated in colorectal cancer tissues, independent of mutations in the KRAS, NRAS, BRAF, PIK3CA, and AKT genes, as well as MSI status. The increased expression of the SIGLEC9 protein in high-grade colorectal cancer tumors appears to shed new light on the potential prognostic significance of this molecule in CRC. SIGLEC9, through its interactions with various cytokines associated with critical processes occurring in the TME, may play a pivotal role in the immunological phenomena observed in CRC, potentially influencing disease progression. The demonstrated associations between SIGLEC9 protein expression and cytokines involved in regulating cell death processes, as well as cytokine and chemokine production, which play a crucial role in shaping the immunosuppressive tumor microenvironment in colorectal cancer, suggest that SIGLEC9 may be considered a promising target for future complex immunotherapeutic approaches in colorectal cancer and other solid tumors, as well as for future clinical studies. For these reasons, SIGLEC9 may represent a promising therapeutic target, particularly in high-grade tumors, regardless of the mutation of the KRAS, NRAS, BRAF, PIK3CA, and AKT genes. A thorough understanding of the impact of targeting SIGLEC9 expression on the immunological properties of tumors, as well as potential clinical outcomes, warrants further investigation into the significance of SIGLEC9 in colorectal cancer.

## Figures and Tables

**Table 1:** Characteristics of the patient group.

	Female	Male	All
Age	67.5 ± 9.77	65 ± 9.03	66 ± 9.37
Tumor localization			
Left-side tumor	28 (68.29%)	35 (76.09%)	63 (72.41%)
Right-side tumor	13 (31.71%)	11 (23.91%)	24 (27.59%)
T parameter			
T1	0 (0%)	4 (8.7%)	4 (4.6%)
T2	10 (24.39%)	5 (10.87%)	15 (17.24%)
T3	27 (65.85%)	31 (67.39%)	58 (66.66%)
T4	4 (9.76%)	6 (13.04%)	10 (11.49%)
N parameter			
N0	19 (46.34%)	22 (47.83%)	41 (47.12%)
N1	15 (36.59%)	17 (36.96%)	32 (36.78%)
N2	7 (17.07%)	7 (15.22%)	14 (16.1%)
M parameter			
M0	37 (90.24%)	37 (80.43%)	74 (85.06%)
M1	4 (9.76%)	9 (19.57%	13 (14.94%)
TNM Stage			
I	8 (19.51%)	7 (15.22%)	15 (17.24%)
II	10 (24.39%)	13 (28.26%)	23 (26.43%)
III	19 (46.34%)	18 (39.13%)	37 (42.53%)
IV	4 (9.76%)	8 (17.39%)	12 (13.79%)
Grading			
High	4 (9.76%)	8 (17.39%)	12 (13.79%)
Low	37 (90.24%)	38 (82.61%)	75 (86.21%)
MSI Status (n = 73)			
MSI-Low	28 (77.78%)	32 (86.49%)	60 (82.19%)
MSI-High	8 (22.22%)	5 (13.51%)	13 (17.81%)
Adjuvant treatment			
Yes	5 (12.2%)	6 (13.04%)	11 (12.64%)
No	36 (87.8%)	40 (86.96%)	76 (87.36%)

**Table 2:** KRAS, NRAS, BRAF, PIK3CA, and AKT gene mutation evaluation details.

Gene	Exon	Amino Acid Change	Nucleotide Change	Cosmic ID
KRAS	2	G12A	c.35G > C	522
		G12D	c.35G > A	521
		G12R	c.34G > C	518
		G12C	c.34G > T	516
		G12S	c.34G > A	517
		G12V	c.35G > T	520
		G13D	c.38G > A	532
	3	A59T	c.175G > A	546
		A59E	c.176C > A	547
		A59G	c.176C > G	28518
		Q61H	c.183A > C	554
		Q61H	c.183A > T	555
		Q61L	c.182A > T	553
		Q61R	c.182A > G	552
	4	K117N	c.351A > C	19940
		K117N	c.351A > T	28519
		K117R	c.350A > G	4696722
		K117E	c.349A > G	-
		A146T	c.436G > A	19404
		A146P	c.436G > C	19905
		A146V	c.437C > T	19900
NRAS	2	G12D	c.35G > A	564
		G12S	c.34G > A	563
		G12C	c.34G > T	562
		G13R	c.37G > C	569
		G13V	c.38G > T	574
	3	A59T	c.175G > A	578
		A59D	c.176C > A	253327
		Q61K	c.181C > A	580
		Q61L	c.182A > T	583
		Q61R	c.182A > G	584
		Q61H	c.183A > C	586
		Q61H	c.183A > T	585
	4	K117R	c.350A > G	-
		A146T	c.436G > A	27174
BRAF	15	V600E	c.1799T > A	476
		V600E2	c.1799-1800TG > AA	-
		V600D	c.1799-1800TG > AT	477
		V600K	c.1798-1799GT > AA	473
PIK3CA	9	E542K	c.1624G > A	760
		E545K	c.1633G > A	763
		E545Q	c.1633G > C	27133
	20	H1047R	c.3140A > G	775
		H1047L	c.3140A > T	776
AKT1	4	E17K	c.49G > A	33765

**Table 3:** Cytokines, chemokines, and growth factor sets assigned to the appropriate Gene Ontology (GO) terms and Kyoto Encyclopedia of Genes and Genomes (KEGG) annotations.

Process Name	Cytokines Involved	Origin
Positive regulation of immune system process	MIF, SCF, MCP1, SDF-1a, VEGFA, MCP3, MCSF, MIP-1a, IL-1a, IL-18, IL-6, RANTES, IL-5, TNF-b, LIF, IL-2, IL-1b, IL-7, IFN-g, IL-13, TNF-a, IL-10, IL-8, IL-4, IP-10, IL-15, IL-2Ra, IL-16, CTACK, IL-12p40, MIP-1b, IL-17	GO
Chemokine signaling pathway	IL-8, MCP1, SDF-1a, GRO-a, IP-10, RANTES, MIP-1a, CTACK, Eotaxin, MCP3, MIP-1b	KEGG
Positive regulation of lymphocyte migration	IP-10, SDF-1a, MIP-1a, CTACK, MIP-1b, MCP3, RANTES	GO
Macrophage chemotaxis	MCP1, IL-8, Eotaxin, MIG, IP-10, GRO-a, MIP-1b, MCP3, RANTES, IL-1b	GO
Regulation of cell population proliferation	CTACK, PDGF-bb, LIF, SCF, MIF, BasicFGF, IFN-g, IL-4, GM-CSF, G-CSF, IL-7, IL-3, MCSF, SDF-1a, SCGF-b, IL-2Ra, TNF-a, IL-6, IL-1b, IL-1a, IP-10, RANTES, IL-5, Eotaxin, IL-2Ra, IL-10, IL-2, IL-18, IL-15, IL-13, IL-9, TNF-b	KEGG
PI3K-Akt signaling pathway	IL-2Ra, bNGF, IL-2, IL-3, IL-4, SCF, MCSF, IFN-a2, HGF, G-CSF, IL-7, PDGF-bb, BasicFGF, IL-6, VEGFA	KEGG
Leukocyte activation	IL-4, IL-15, IFN-g, SCF, IL-2Ra, IL-8, MCSF, IL-13, IL-18, MIP-1a, RANTES, IL-10, GM-CSF, IL-9, IL-7, IFN-a2, IL-2, IL-6, TNF-a	GO
Inflammatory response	IL-9, CTACK, Eotaxin, MCP1, IFN-a2, IL-1Ra, IL-2Ra, IFN-g, IL-15, IL-1a, IL-6, IL-17, IL-4, MCP3, MIP-1a, IL-18, CTACK, MIF, TNF-a, RANTES, MCSF, MIG, IL-1b, IL-5, IL-10, IL-8, IL-13, IP-10, MIP-1b	GO
Regulation of cell death	VEGFA, HGF, SDF-1a, bNGF, BasicFGF, SCF, IL-6, IL-7, G-CSF, IL-1a, IL-4, MCSF, IL-13, MIP-1a, IP-10, IL-1b, IL-9, IL-2, IFN-g, MCP1, TNF-b, GM-CSF, TNF-a, RANTES, IL-10, TNF-a, MIF	GO
RAF/MAP kinase cascade	PDGF-bb, IL-5, IL-2, BasicFGF, IL-2Ra, SCF, GM-CSF, HGF, IL-3	KEGG
Positive regulation of cytokine production	IL-9, IL-12p70, GM-CSF, IL-10, HGF, IL-2, IL-15, IL-1b, IL-18, IFN-g, IL-7, IL-4, TNF-a, IL-17, MIF, TNF-b, IL-16, IL-13, MIP-1a, IL-1a, IL-6	GO

MIF—Macrophage Migration Inhibitory Factor; SCF—Stem Cell Factor; MCP1—Monocyte Chemoattractant Protein 1; SDF-1a—stromal cell-derived factor 1 alpha; VEGFA—Vascular Endothelial Growth Factor A; MCP3—Monocyte Chemoattractant Protein 3; MCSF—Macrophage Colony-Stimulating Factor; MIP-1a—Macrophage Inflammatory Protein 1 alpha; IL-1a—Interleukin 1 alpha; IL-18—Interleukin 18; IL-6—Interleukin 6; RANTES—Regulated on Activation, Normal T Cell Expressed and Secreted; IL-5—Interleukin 5; TNF-b—Tumor Necrosis Factor beta; IL-2—Interleukin 2; IL-1b—Interleukin 1 beta; IL-7—Interleukin 7; IFN-g—Interferon gamma; IL-13—Interleukin 13; TNF-a—Tumor Necrosis Factor alpha; IL-10—Interleukin 10; IL-8—Interleukin 8; IL-4—Interleukin 4; IP-10—Interferon Gamma-Induced Protein 10; IL-15—Interleukin 15; IL-2Ra—Interleukin 2; Receptor alpha CTACK—Cutaneous T Cell-Attracting Chemokine; IL-12p40—Interleukin 12 p40; MIP-1b—Macrophage Inflammatory Protein 1 beta; IL-17—Interleukin 17; GRO-a—Growth-Regulated Oncogene alpha Eotaxin—Eotaxin (CCL11); MIG—Monokine Induced by Gamma Interferon; PDGF-bb—Platelet-Derived Growth Factor BB; BasicFGF—Basic Fibroblast Growth Factor; GM-CSF—granulocyte-macrophage colony-stimulating factor; G-CSF—Granulocyte Colony-Stimulating Factor; IL-3—Interleukin 3; IL-9—Interleukin 9.

**Table 4:** SIGLEC9 protein concentration and TNM scale parameters, tumor stage, and TILs. Kendall’s Tau rank correlation coefficient *p*-value and Tau for T and N parameters, tumor stage, and TILs for SIGLEC9 expression. *p*-value for the M parameter was derived from the Mann–Whitney U test for SIGLEC9 expression.

	T Parameter		N Parameter		M Parameter	Tumor Stage		Tumor-Infiltrated Lymphocytes (TILs)	
	*p*-Value	Tau	*p*-Value	Tau	*p*-Value	*p*-Value	Tau	*p*-Value	Tau
SIGLEC9	0.6189	−0.0423	0.6165	−0.042	0.8302	0.9969	−0.00032	0.3484	−0.0850

**Table 5:** Frequencies of mutations in KRAS, NRAS, BRAF, PIK3CA, and AKT genes in the studied cohort.

Gene	Mutation Status		Percent (%)	
n = 69	Wild-Type	Mutant	Percent Among Mutation of the One Gene	Percent Among All Group
KRAS	45	24		34.78%
KRAS-117-STATUS	65	4	16.67%	5.79%
KRAS-12/13-STATUS	53	16	66.67%	23.19%
KRAS-59-STATUS	66	3	12.50%	4.35%
KRAS-146-STATUS	67	2	8.33%	2.90%
KRAS-61-STATUS	67	2	8.33%	2.90%
NRAS	58	11		15.94%
NRAS-12-13-STATUS	63	6	54.54%	8.69%
NRAS-61-STATUS	64	5	45.45%	7.25%
PIK3CA	63	6		8.69%
PIK3CA 542/545	64	5	83.33%	7.25%
PIK3CA 1047	68	1	16.67%	1.45%
BRAF	64	5		7.25%
AKT	68	1		1.45%

**Table 6:** Eigenvalue and the percentage of explained variance for three factors (principal components) from the PCA for positive regulation of lymphocyte chemotaxis with SIGLEC9 protein expression.

	Eigenvalue	Variance (%)	Cumulative Variance (%)
PCA Factor 1	3.22847925	64.569585	64.56958
PCA Factor 2	1.24083953	24.816791	89.38638
PCA Factor 3	0.30147199	6.029440	95.41582

**Table 7:** Eigenvalue and the percentage of explained variance for three factors (principal components) from the PCA for PI3K-Akt signaling pathway with SIGLEC9 protein expression.

	Eigenvalue	Variance (%)	Cumulative Variance (%)
PCA Factor 1	7.033718538	54.1055272	54.10553
PCA Factor 2	2.213658319	17.0281409	71.13367
PCA Factor 3	1.134435018	8.7264232	79.86009

**Table 8:** Eigenvalue and the percentage of explained variance for three factors (principal components) from the RAF/MAP kinase cascade set of cytokines with SIGLEC9 protein expression.

	Eigenvalue	Variance (%)	Cumulative Variance (%)
PCA Factor 1	4.84814423	53.8682693	53.86827
PCA Factor 2	2.00451770	22.2724189	76.14069
PCA Factor 3	0.79595388	8.8439320	84.98462

**Table 9:** Eigenvalue and the percentage of explained variance for three factors (principal components) from the PCA for regulation of cell death with SIGLEC9 protein expression.

	Eigenvalue	Variance (%)	Cumulative Variance (%)
PCA Factor 1	10.82112	43.28449	43.28449
PCA Factor 2	4.25614	17.02457	60.30906
PCA Factor 3	2.90319	11.61277	71.92183

**Table 10:** Eigenvalue and the percentage of explained variance for three factors (principal components) from the PCA for positive regulation of cytokine production with SIGLEC9 protein expression.

	Eigenvalue	Variance (%)	Cumulative Variance (%)
PCA Factor 1	6.943532047	40.84430616	40.84431
PCA Factor 2	3.265741685	19.21024521	60.05455
PCA Factor 3	1.712493249	10.07348970	70.12804

**Table 11:** Loadings of three factors (PCA factors) after varimax rotation. Coordinates for the variables for positive regulation of lymphocyte chemotaxis with SIGLEC9 protein expression in PCA.

Variable	Factor 1	Factor 2	Factor 3
Positive Regulation of Lymphocyte Chemotaxis			
RANTES	0.9017337	−0.2391467	−0.1393645
MIP-1a	0.8323022	−0.4067699	−0.2910296
CTACK	0.8196023	0.5399496	−0.0425685
MCP3	0.6889986	0.6992168	0.1138596
MIP-1b	0.7590531	−0.4875827	0.4272882

**Table 12:** Loadings of three factors (PCA factors) after varimax rotation. Coordinates for the variables for PI3K-Akt signaling pathway with SIGLEC9 protein expression in PCA.

Variable	Factor 1	Factor 2	Factor 3
PI3K-Akt Signaling Pathway			
IL-2Ra	0.8299376	0.1253971	−0.273751496
bNGF	0.7921063	0.2638864	0.369706577
IL-2	0.8072090	0.3931289	0.280055095
IL-3	0.9100592	0.3145049	0.009026208
IL-4	0.8866259	0.1873077	−0.268366930
SCF	0.7297464	−0.4156044	−0.108076451
IFN-a2	0.9408995	0.1564561	−0.123488217
HGF	0.4488180	−0.6992193	0.227174393
G-CSF	0.6059206	−0.6363815	−0.067589068
IL-7	0.6101636	−0.4744356	0.270884156
PDGF-bb	0.7687188	0.3428542	−0.420590634
IL-6	0.5314466	−0.5903031	−0.138248068
VEGFA	0.4685372	0.2399159	0.647908531

**Table 13:** Loadings of three factors (PCA factors) after varimax rotation. Coordinates for the variables for RAF/MAP kinase cascade set of cytokines with SIGLEC9 protein expression in PCA.

Variable	Factor 1	Factor 2	Factor 3
RAF/MAP Kinase Cascade			
PDGF-bb	0.8478336	−0.12511962	−0.37707867
IL-5	0.6425866	−0.57636450	0.45452259
IL-2	0.8908608	−0.09032555	0.38307124
BasicFGF	0.4735157	−0.62287655	−0.37468967
IL-2Ra	0.8257607	0.06446405	−0.31885016
SCF	0.5897755	0.70414056	−0.13664277
GM-CSF	0.8359521	0.28703620	0.07410667
HGF	0.2780528	0.81867165	0.14824862
IL-3	0.9448330	−0.08916412	0.11060162

**Table 14:** Loadings of three factors (PCA factors) after varimax rotation. Coordinates for the variables for regulation of cell death set of cytokines with SIGLEC9 protein expression in PCA.

Variable	Factor 1	Factor 2	Factor 3
Regulation of Cell Death			
VEGFA	0.4840667	0.25002984	−0.09154334
HGF	0.4238375	−0.62106271	0.46756715
SDF-1a	0.9643265	−0.06391033	0.11602199
BasicFGF	0.0126641	0.60963482	−0.01701672
SCFIL-6	0.7296873	−0.24906796	0.33561221
IL-7	0.5617344	−0.36844819	0.15402731
G-CSF	0.6733436	−0.23122607	0.32902755
IL-1a	0.8219997	−0.48688632	−0.12599496
IL-4	0.5153230	0.59465365	0.28639514
MCSF	0.7904213	0.35301541	0.12831741
IL-13	0.4894104	0.68555729	−0.25135616
MIP-1a	0.3852280	−0.62036979	0.38245010
IP-10	0.7433359	−0.48001001	−0.31316698
IL-1b	0.7006524	0.02405145	−0.54291777
IL-9	0.2778055	0.68147115	−0.32197988
IL-2	0.9483551	−0.03989836	−0.25204134
IFN-g	0.5677005	0.45562259	0.52446682
MCP1	0.2519262	0.57833071	0.07481690
TNF-b	0.7488402	−0.05509508	−0.50052492
GM-CSF	0.9077088	−0.01872117	−0.38056117
TNF-a	0.5831471	0.04522961	0.45376126
RANTES	0.8702113	0.32243854	0.26805892
IL-10	0.8203877	−0.11867462	−0.49377048
SDF-1a	0.7984569	0.20187821	0.40966064
MIF	0.1529922	−0.45552068	−0.41058894

**Table 15:** Loadings of three factors (PCA factors) after varimax rotation. Coordinates for the variables for positive regulation of cytokine production set of cytokines with SIGLEC9 protein expression in PCA.

Variable	Factor 1	Factor 2	Factor 3
Positive Regulation of Cytokine Production			
IL-9	0.90873375	0.045840154	0.2718711
GM-CSF	0.64419740	0.122305699	−0.4609939
HGF	0.41349773	0.692268020	−0.3881720
IL-15	0.67154792	−0.281675081	−0.3865051
IL-1b	0.31225075	−0.682321106	0.4503141
IL-18	0.85351774	0.109580592	0.1935392
IL-7	0.66485202	0.195094700	−0.1276783
IL-4	0.81642923	−0.243670628	−0.1505702
TNF-a	0.91517726	−0.242655203	−0.2127553
IL-17	0.55334703	−0.250268274	0.1940640
MIF	0.09202063	0.465882207	0.5414278
TNF-b	0.86147721	0.007597468	0.4126536
IL-16	0.24982133	0.746659954	−0.2761531
MIP-1a	0.67327028	0.457895739	0.3882208
IL-1a	0.56788096	−0.601632157	−0.2280433
IL-6	0.59867134	0.471727925	0.0404402

## Data Availability

GSEA data: Dampier CH (2020). “FieldEffectCrc: Tumor, tumor-adjacent normal, and healthy colorectal transcriptomes as SummarizedExperiment objects.” https://bioconductor.org/packages/release/data/experiment/html/FieldEffectCrc.html (accessed on 15 November 2022). Experimental data can be shared upon request.
